# Sphingolipids in prostate cancer prognosis: integrating single-cell and bulk sequencing

**DOI:** 10.18632/aging.205803

**Published:** 2024-05-06

**Authors:** Shan Zhou, Li Sun, Fei Mao, Jing Chen

**Affiliations:** 1 Department of Ultrasound, The Affiliated Huaian No. 1 People’s Hospital of Nanjing Medical University, Huaian City 223300, People’s Republic of China; 2 Department of Urology, The Affiliated Huaian No. 1 People’s Hospital of Nanjing Medical University, Huaian City 223300, People’s Republic of China; 3 Department of Urology, The Affiliated Huaian No. 1 People’s Hospital of Xuzhou Medical University, Huaian City 223300, People’s Republic of China

**Keywords:** prostate cancer, sphingolipid metabolism, tumor microenvironment, prognosis, single-cell sequencing

## Abstract

Background: Stratifying patient risk and exploring the tumor microenvironment are critical endeavors in prostate cancer research, essential for advancing our understanding and management of this disease.

Methods: Single-cell sequencing data for prostate cancer were sourced from the pradcellatlas website, while bulk transcriptome data were obtained from the TCGA database. Dimensionality reduction cluster analysis was employed to investigate heterogeneity in single-cell sequencing data. Gene set enrichment analysis, utilizing GO and KEGG pathways, was conducted to explore functional aspects. Weighted gene coexpression network analysis (WGCNA) identified key gene modules. Prognostic models were developed using Cox regression and LASSO regression techniques, implemented in R software. Validation of key gene expression levels was performed via PCR assays.

Results: Through integrative analysis of single-cell and bulk transcriptome data, key genes implicated in prostate cancer pathogenesis were identified. A prognostic model focused on sphingolipid metabolism (SRSR) was constructed, comprising five genes: “FUS,” “MARK3,” “CHTOP,” “ILF3,” and “ARIH2.” This model effectively stratified patients into high-risk and low-risk groups, with the high-risk cohort exhibiting significantly poorer prognoses. Furthermore, distinct differences in the immune microenvironment were observed between these groups. Validation of key gene expression, exemplified by ILF3, was confirmed through PCR analysis.

Conclusion: This study contributes to our understanding of the role of sphingolipid metabolism in prostate cancer diagnosis and treatment. The identified prognostic model holds promise for improving risk stratification and patient outcomes in clinical settings.

## INTRODUCTION

Prostate cancer ranks among the most prevalent cancers affecting men globally [1]. Its risk escalates significantly with age, being infrequent in men below 40 but increasingly prevalent among those over 65 years old [2]. Despite considerable progress in prostate cancer treatment, substantial limitations and challenges persist, contingent on cancer stage, aggressiveness, and patient variability. Notably, when tumor cells metastasize beyond the prostate, treatment options become more restricted. While hormone therapy and chemotherapy can help manage the disease, they often fall short of a cure, with the cancer eventually developing resistance to these treatments. In particular, some patients may experience resistance to hormone therapy (androgen deprivation therapy), evolving into castration-resistant prostate cancer (CRPC) [3]. Although new drugs have emerged to address this issue, managing CRPC remains arduous, with limited treatment choices. Consequently, an urgent imperative exists to formulate fresh patient risk stratification methodologies, explore the tumor microenvironment in prostate cancer patients, and pinpoint novel, precise biomarkers. This endeavor aims to facilitate early diagnosis, early intervention, and enhanced patient prognosis.

Metabolic reprogramming within the tumor microenvironment represents a complex and pivotal phenomenon in cancer biology, exerting influence across all facets of cancer progression and behavior [4]. A primary role of this metabolic reprogramming in the tumor microenvironment revolves around facilitating the rapid and unbridled proliferation of cancer cells. These cells demand a continuous supply of energy and the fundamental constituents necessary for DNA, RNA, and protein synthesis. Even within environments marked by nutritional constraints, cancer cells exhibit metabolic adaptability, enabling them to shift their metabolic pathways in favor of generating these essential molecules [5]. Cancer cells adeptly manipulate their metabolism to elude the immune system’s surveillance. Through metabolic reprogramming, they modify their nutrient utilization and produce immunosuppressive metabolites. This results in the creation of an immunosuppressive microenvironment that impedes the immune system’s ability to detect and eradicate cancer cells. Consequently, metabolic reprogramming is intricately linked to cancer cell invasion and metastasis. Metabolic alterations can additionally augment cancer cells’ capacity to degrade the extracellular matrix, migrate through tissues, and establish secondary tumors in distant organs [6]. Tumors frequently contain regions with low oxygen levels (hypoxia) due to inadequate blood supply. Cancer cells can adapt to such conditions through metabolic reprogramming, switching to alternative metabolic pathways like glycolysis (the Warburg effect). This metabolic shift induces angiogenesis, promoting the formation of new blood vessels. Furthermore, metabolic reprogramming can confer resistance to various cancer treatments, including chemotherapy and targeted therapies. Cancer cells can develop mechanisms that reduce their reliance on specific metabolic pathways targeted by drugs.

The tumor microenvironment constitutes an intricate ecosystem comprising not only cancer cells but also a diverse array of stromal cells, such as fibroblasts, immune cells, and endothelial cells [7]. Metabolic reprogramming in cancer cells can impact the metabolism of stromal cells, and conversely, stromal cell metabolism can influence cancer cell behavior, thus collectively shaping the tumor’s overall behavior. This comprehension of metabolic alterations within the tumor microenvironment has paved the way for the identification of potential therapeutic targets.

Researchers are actively investigating drugs designed to disrupt specific metabolic pathways or restore normalcy to the metabolic milieu within cancer cells. Such efforts aim to enhance the efficacy of cancer treatments. Moreover, metabolic changes within tumors hold substantial potential as diagnostic and prognostic biomarkers. The analysis of tumor metabolic characteristics can facilitate the classification of cancer subtypes, prognosticate patient outcomes, and steer personalized treatment strategies.

Sphingolipid metabolism assumes a pivotal role within the tumor microenvironment, exerting influence over all facets of tumor progression and therapeutic responsiveness. Sphingolipids, a lipid class encompassing sphingolipins, ceramides, and sphingosine-1-phosphate (S1P), actively participate in cell signaling, proliferation, survival, and migration [8–11]. Ceramide, a prominent sphingolipid, emerges as a pro-apoptotic molecule that fosters programmed cell death [12]. Elevated ceramide levels can trigger apoptosis in cancer cells [13]. Conversely, diminished ceramide levels and heightened S1P levels within the tumor microenvironment foster cancer cell survival by inhibiting apoptosis [14]. Notably, S1P has been demonstrated to stimulate angiogenesis, the process of generating new blood vessels essential for tumor growth and metastasis [15]. S1P effectively promotes endothelial cell migration, blood vessel formation, and enhances nutrient supply to tumors [16]. Sphingolipids also wield influence over the functionality of immune cells in the tumor microenvironment [17]. Ceramide can prompt apoptosis of immune cells and potentially hinder the anti-tumor immune response. In contrast, S1P can attract immune cells to the tumor site, thereby influencing the balance between immunosuppression and activation within the tumor microenvironment. Signaling through the sphingosine-1-phosphate receptor (S1PR) can further stimulate the migration and invasion of tumor cells. S1P serves as a chemical attractant, guiding cancer cells to metastasize into blood and lymphatic vessels, thus facilitating their dissemination to distant organs [17].

Modifying sphingolipid metabolism can bolster the chemotherapy resistance of cancer cells. Elevated ceramide levels sensitize cancer cells to apoptosis induced by chemotherapy, whereas heightened S1P levels bolster drug resistance by fortifying cell survival pathways [9]. Sphingolipids exert regulatory control over inflammation within the tumor microenvironment, with ceramides often linked to pro-inflammatory responses, while S1P may exhibit either pro-inflammatory or anti-inflammatory effects contingent on the microenvironment. Sphingolipids also form components of extracellular vesicles (EVs), including exosomes, that partake in intercellular communication within the tumor microenvironment. These vesicles are capable of transferring bioactive lipids, including S1P, among cells, thereby influencing tumor growth and metastasis [10].

Presently, targeting sphingolipid metabolism has emerged as a prospective therapeutic approach in cancer treatment [18–20]. The development of drugs capable of modulating sphingolipid levels or disrupting sphingolipid signaling pathways holds potential for controlling tumor growth and sensitizing cancer cells to treatment [11]. In summary, sphingolipid metabolism occupies a multifaceted role within the tumor microenvironment, impacting various facets of tumor biology encompassing cell survival, angiogenesis, immune modulation, migration, and treatment response. A thorough comprehension of these processes at the molecular level could furnish invaluable insights for the development of novel cancer therapies and the improvement of treatment outcomes.

However, the precise significance of sphingolipid metabolism within the prostate cancer microenvironment remains uncertain. In our study, we conducted multi-omics analyses, leveraging both single-cell sequencing techniques and bulk transcriptome analysis approaches, to elucidate the relevance of sphingolipid metabolism in prostate cancer. Concurrently, we established an associated prognostic model. Our findings hold the potential to serve as a reference for evaluating the prognosis of prostate cancer.

## MATERIALS AND METHODS

### Single-cell sequencing data processing

The research made use of the website (http://www.pradcellatlas.com/#/) to obtain single-cell data related to prostate cancer, comprising a dataset encompassing 13 samples of single-cell sequencing data [21]. To ensure data quality, several criteria were applied:

1. Genes expressed in fewer than 3 cells were excluded.
2. Cells with gene expression levels falling between 200 and 5000 were retained.
3. Cells with less than 10 percent of their mitochondrial genes were retained.
4. Cells with a total gene expression below 50000 were preserved.

Following these quality control measures, steps were taken to mitigate batch effects between samples and cells using the “SCT” approach. To reduce the dimensionality of the samples, the “dims” parameter was set to 20, and the UMAP method was employed. The parameters were configured with K.Paam set at 20, resolution at 0.4, a random seed of 2023, and the KNN method utilized for cell clustering. Cell annotations were determined based on marker genes corresponding to cell types documented in the published literature.

### Bulk transcriptome data processing

The prostate cancer TCGA cohort data were acquired from the UCSC Xena website (https://xenabrowser.net/). These data underwent a standardized processing procedure, which included the alignment of transcriptome data with prognostic clinical data. Samples containing both types of data were retained for subsequent analysis.

### Acquisition of gene set related to sphingolipid metabolism

Sphingolipid metabolism-related genes were sourced from the GeneCard website (https://www.genecards.org/). Genes with scores exceeding 3 were selected for further analysis, resulting in the retention of a total of 118 genes.

### GO enrichment analysis

The GO database encompasses three components: cellular component (CC), molecular function (MF), and biological process (BP). To investigate enriched biological functions, we utilized the GO database and compared the provided gene list with the gene distribution within each category of biological functions. This step uses the clusterProfiler R package.

### KEGG enrichment analysis

We utilized the KEGG database to identify enriched pathways associated with the given gene list. This involved a matching process where we compared the provided gene list with the genes documented in the human pathways within the KEGG database. This step uses the clusterProfiler R package.

### Weighted gene coexpression network analysis (WGCNA)

In this study, we applied WGCNA (Weighted Gene Co-expression Network Analysis) to explore gene sets closely associated with sphingolipid metabolism in prostate cancer bulk-seq data. To determine the appropriate soft threshold, we considered a range from 1 to 10 with increments of 1, and from 12 to 20 with increments of 2, using the “pickSoftThreshold” function from the “WGCNA” R package. We set the minimum number of genes per module to 150, configured a “deepSplit” value of 2 for gene clustering, and integrated the analysis with sphingolipid phenotype data.

### Construction and validation of the prognostic model

In this study, we divided the TCGA cohort into two groups randomly using the “caret” package. Initially, we conducted univariate Cox analysis, with a significance threshold set at *p* < 0.05, to identify genes associated with prognosis. Subsequently, we utilized LASSO regression for further screening and the construction of a prognostic model. We then proceeded to perform prognostic analysis of the model in three separate sets: the training set, the validation set, and the entire cohort, to comprehensively evaluate its predictive performance.

### Analysis of immune cell infiltration

We utilized the TIMER2 website (http://timer.cistrome.org/) to acquire immune cell infiltration data from the TCGA database, which had been derived using diverse computational techniques. Differences in the distribution of immune cells between the high-risk and low-risk groups determined by the model were subsequently examined. To visualize these distinctions, heat maps were employed. Following this, the expression levels of immunodetection point genes, leukocyte antigens, and tumor necrosis-related genes between the two groups were investigated.

### Drug sensitivity analysis

The “pRRophetic” package was employed in this study to predict the IC50 (half-maximal inhibitory concentration) of drugs. This prediction involved a comparison between the expression pattern of prostate cancer and the training pattern linked to drug responses.

### The construction of the nomogram

To evaluate patient outcomes, we utilized the “regplot” package to generate nomograms, which are based on patient models and clinical data. These nomograms provide a comprehensive visualization and assessment of patient prognostic information.

### The expression of model gene ILF3 in prostate cancer was verified by PCR

Five patients with prostate cancer were enrolled between January 2022 and December 2022, all of whom were diagnosed with prostate cancer by prostate biopsy before surgery. After admission, they underwent surgery to remove prostate cancer. The tumor tissue was divided into an experimental group, and the adjacent normal tissue was used as normal control group. The expression level of ILF3 was detected by PCR. The study was approved by the Ethics Committee of The Affiliated Huaian No. 1 People’s Hospital of Nanjing Medical University. Tissue-derived total RNA was extracted and utilized to synthesize complementary DNA (cDNA) following the manufacturer’s instructions provided in the PrimeScript^™^ RT reagent kit with gDNA Eraser (TaKaRa, Kusatsu, Japan). Quantitative reverse transcription-polymerase chain reaction (qRT-PCR) was conducted using the AceQ Universal SYBR qPCR Master Mix (Vazyme, Nanjing, China) on a QuantStudio 7 PCR system (Thermo Fisher Scientific, CA, USA). The primer set employed in this study is detailed below:

For ILF3: Forward Primer for ILF3: 5′-GAACGTA AAACAGCAGGGGC-3′; Reverse Primer for ILF3: 5′-GTCCATCCACTTCGACCTCC-3′.

For GAPDH: Forward Primer: 5′-ACCCACTCCTCCA CCTTTGA-3′; Reverse Primer: 5′-CTGTTGCTGTAG CCAAATTCGT-3′.

## RESULTS

### Single-cell sequencing data analysis of sphingolipid metabolism in prostate cancer

**Figure 1:**
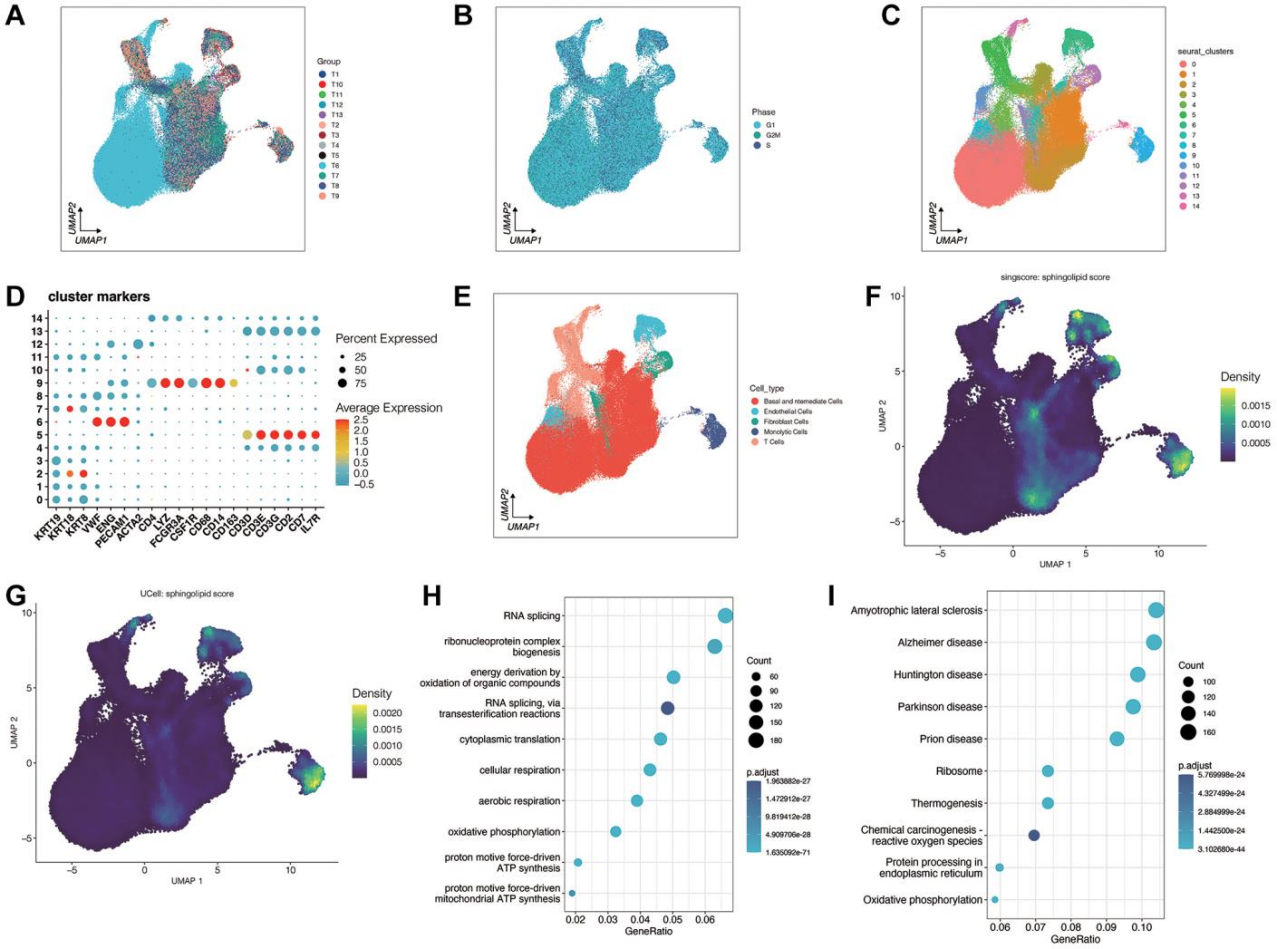
**Analysis of single-cell sequencing data for prostate cancer.** (**A**, **B**) Quality control and sample integration. (**C**–**E**) The dimensionality reduction clustering was performed on all cells. All cells are grouped into 15 clusters. After annotating the different clusters, a total of five cell types were obtained. (**F**, **G**) A sphingolipid metabolism score was performed. (**H**, **I**) Gene enrichment analysis between high sphingolipid metabolism score group and low sphingolipid metabolism score group.

Initially, our investigation centered on sphingolipid metabolism enrichment at the single-cell level. In Figure 1A, 1B, it is evident that following quality control and sample integration, we retained 11 tumor samples, and there was no noticeable batch effect among them. The clustering of tumor samples into 15 distinct clusters is demonstrated in Figure 1C–1E. We categorized these clusters into 5 cell types based on the expression of cell type marker genes from previously published research. These cell types include Basal and Intermediate Cells, Endothelial Cells, Fibroblast Cells, Monocytic Cells, and T Cells.

Subsequently, we calculated sphingolipid metabolic enrichment scores utilizing the “UCell” and “singscore” methods from the “irGSEA” package, using sphingolipid metabolic genes. Patients were then divided into high and low groups based on the median value of metabolic enrichment scores, using the “singscore” method. Differential expression analysis was performed, leading to the identification of 3014 genes with a significance threshold of *p* < 0.05. As depicted in Figure 1F, 1G, the enrichment results from both methods were consistent, with sphingolipid metabolism primarily enriched in Endothelial Cells and Fibroblast Cells.

Subsequent analysis focused on pathways potentially associated with sphingolipid metabolism, as shown in Figure 1H, 1I. These pathways predominantly encompass RNA synthesis, ribonucleoprotein complex synthesis, energy synthesis, heat production, and endoplasmic reticulum protein processing of organic compound oxidation.

### WGCNA analysis

**Figure 2:**
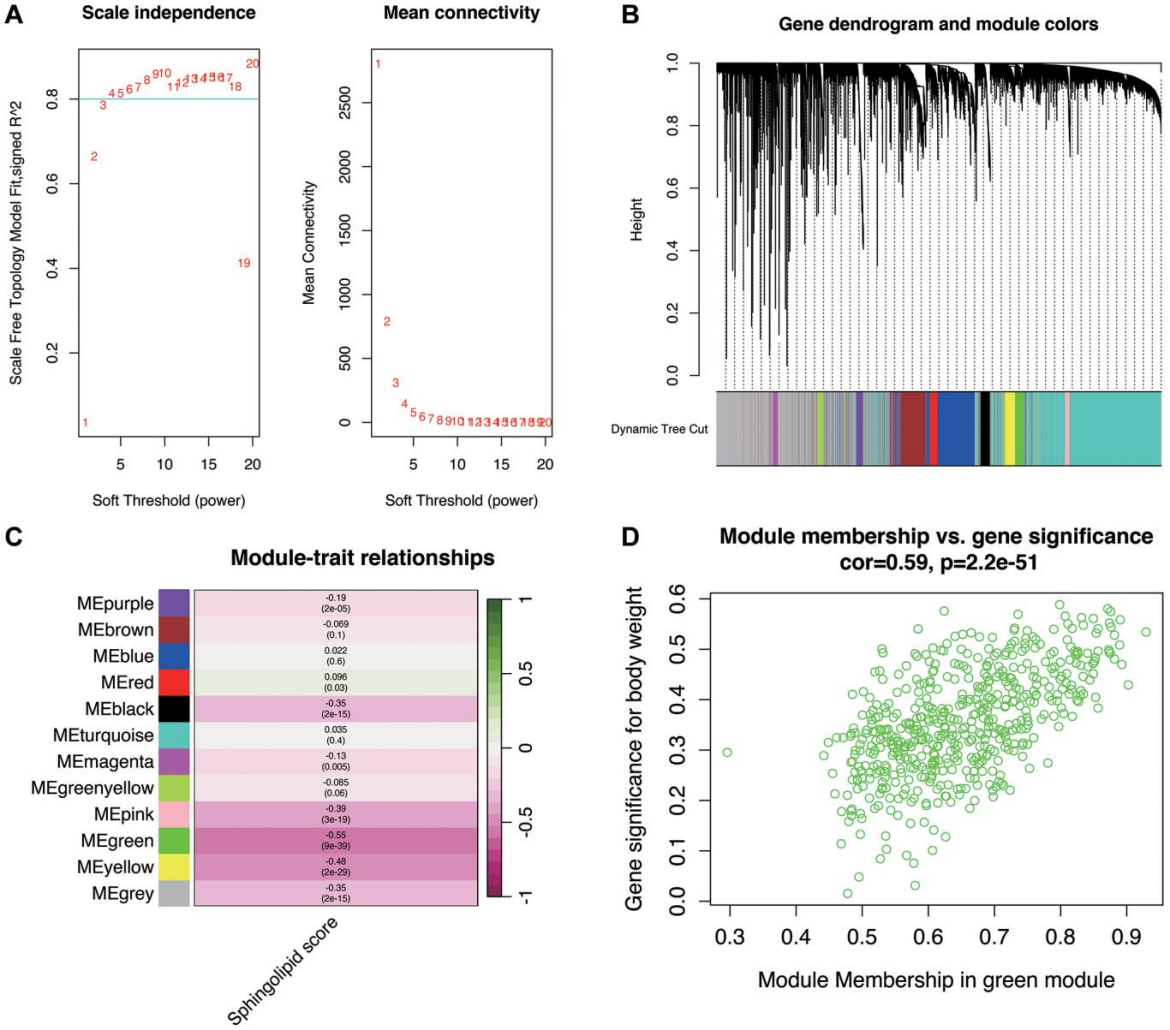
**Weighted gene coexpression network analysis.** (**A**) When the optimal soft threshold is 7, the data conform to the power law distribution, and the Mean connectivity tends to be stable. (**B**, **C**) Genes clusters. They were clustered into 11 non-gray modules, of which the green module was most correlated with the sphingolipid metabolism phenotype. (**D**) Correlation of green modules.

Following that, we conducted a transcriptome data analysis to pinpoint genes linked to sphingolipid metabolism. Initially, we determined the sphingolipid metabolism enrichment fraction for each prostate cancer sample using the ssGSEA method. Subsequently, we carried out WGCNA analysis. In Figure 2A, it’s evident that when the optimal soft threshold is set to 7, the data conforms to a power law distribution, and the Mean connectivity shows stability.

In Figure 2B, 2C, the genes were grouped into 11 non-gray modules, with the green module demonstrating the highest correlation with the sphingolipid metabolism phenotype (correlation = −0.55 and *p* < 0.05). A closer look at the inter-gene correlations within the green module is presented in Figure 2D. Module membership within the green module was found to be positively correlated with Gene significance for body weight (correlation = 0.59 and *p* < 0.05).

### Construction and validation of the prognostic model

To identify more robust genes associated with sphingolipid metabolism in prostate cancer, we merged the 3014 genes obtained from single-cell analysis with the 534 genes acquired from WGCNA analysis, resulting in a total of 57 genes. For clinical applications, we constructed a prognostic model.

**Figure 3:**
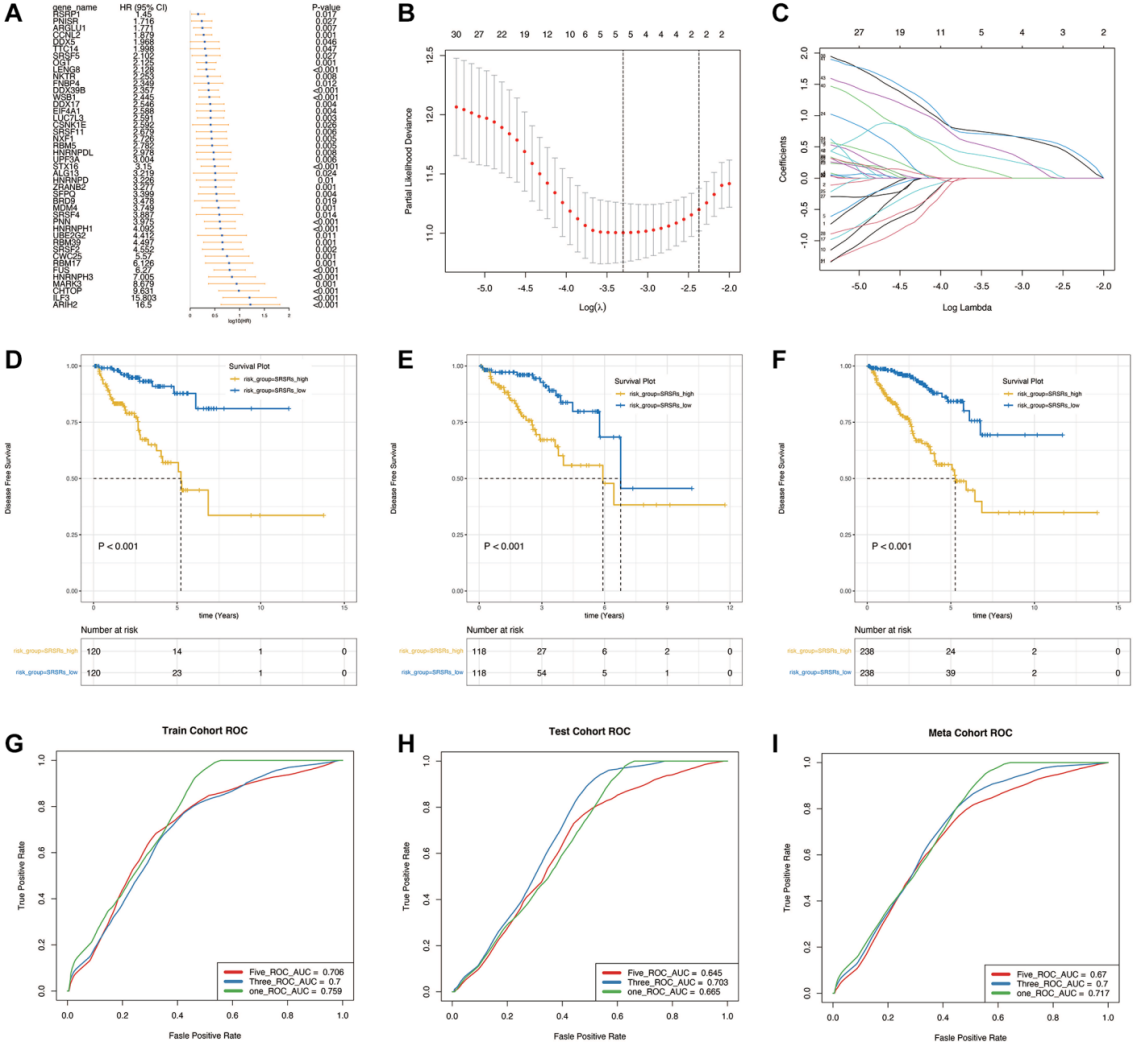
**Construction and validation of the prognostic model.** (**A**) Univariate Cox analysis to obtain the prognosis-related genes. (**B**, **C**) LASSO regression to construct the prognostic model. (**D**–**F**) Survival analysis in the training cohort, validation cohort and the entire meta cohort. (**G**–**I**) The prognostic ROC analysis in the training cohort, validation cohort and the entire meta cohort.

In Figure 3A, we conducted a univariate Cox analysis with a significance threshold of *p* < 0.05, and this led to retaining a total of 43 genes. Subsequently, we employed LASSO regression, as shown in Figure 3B, 3C, which resulted in the selection of 5 genes for the model: “FUS,” “MARK3,” “CHTOP,” “ILF3,” and “ARIH2.” The sphingolipid-related risk score (SRSRs) was then computed based on the sphingolipid metabolism correlation model.

Figure 3D–3F illustrate that in the training cohort, validation cohort, and the entire meta cohort, patients in the SRSRs_high group exhibited poorer prognosis and shorter disease-free survival. Prognostic ROC analysis, as depicted in Figure 3G–3I, indicated that the model demonstrated good accuracy in assessing patient prognosis at 1, 3, and 5 years.

### The expression of model genes in different cell types and pseudo-time series analysis

**Figure 4:**
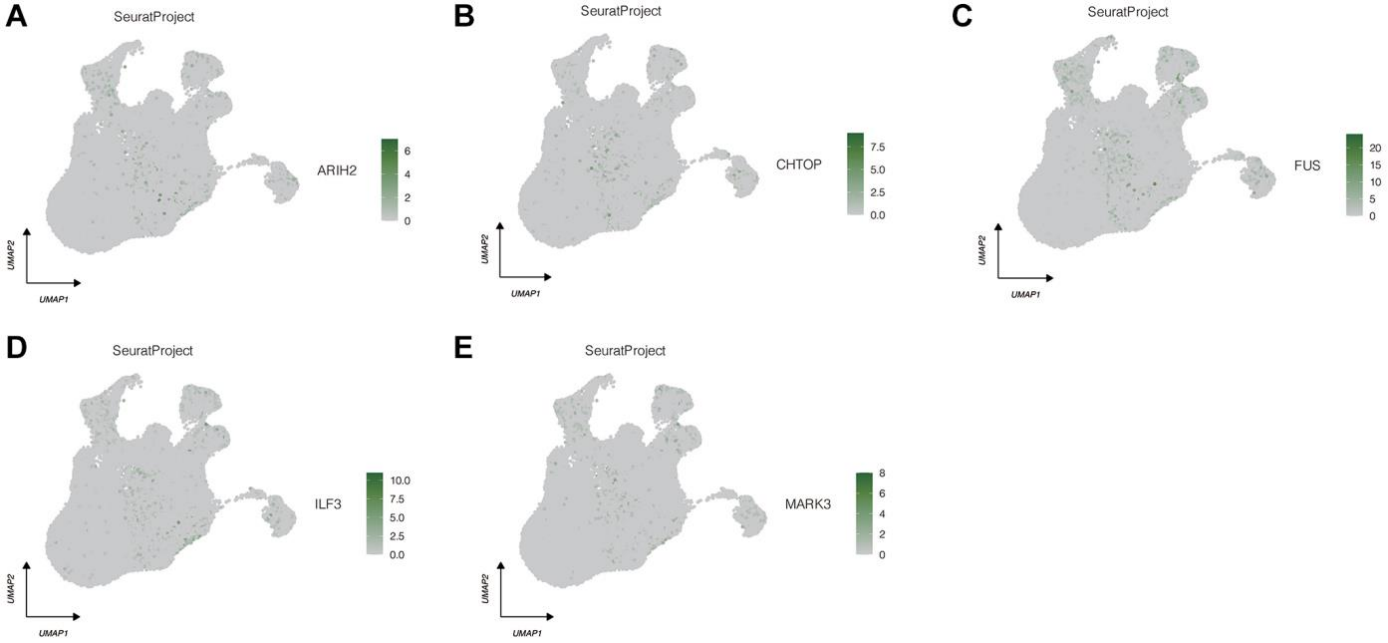
**Expression localization of 5 model genes in different cell types.** (**A**) ARIH2. (**B**) CHTOP. (**C**) FUS. (**D**) ILF3. (**E**) MARK3.

We proceeded with the analysis of the expression distribution of the five genes comprising the model at the single-cell level. As shown in Figure 4A–4E, these five genes predominantly exhibit expression in Fibroblast Cells. Consequently, we isolated the Fibroblast Cells and conducted a pseudo-time series analysis.

**Figure 5:**
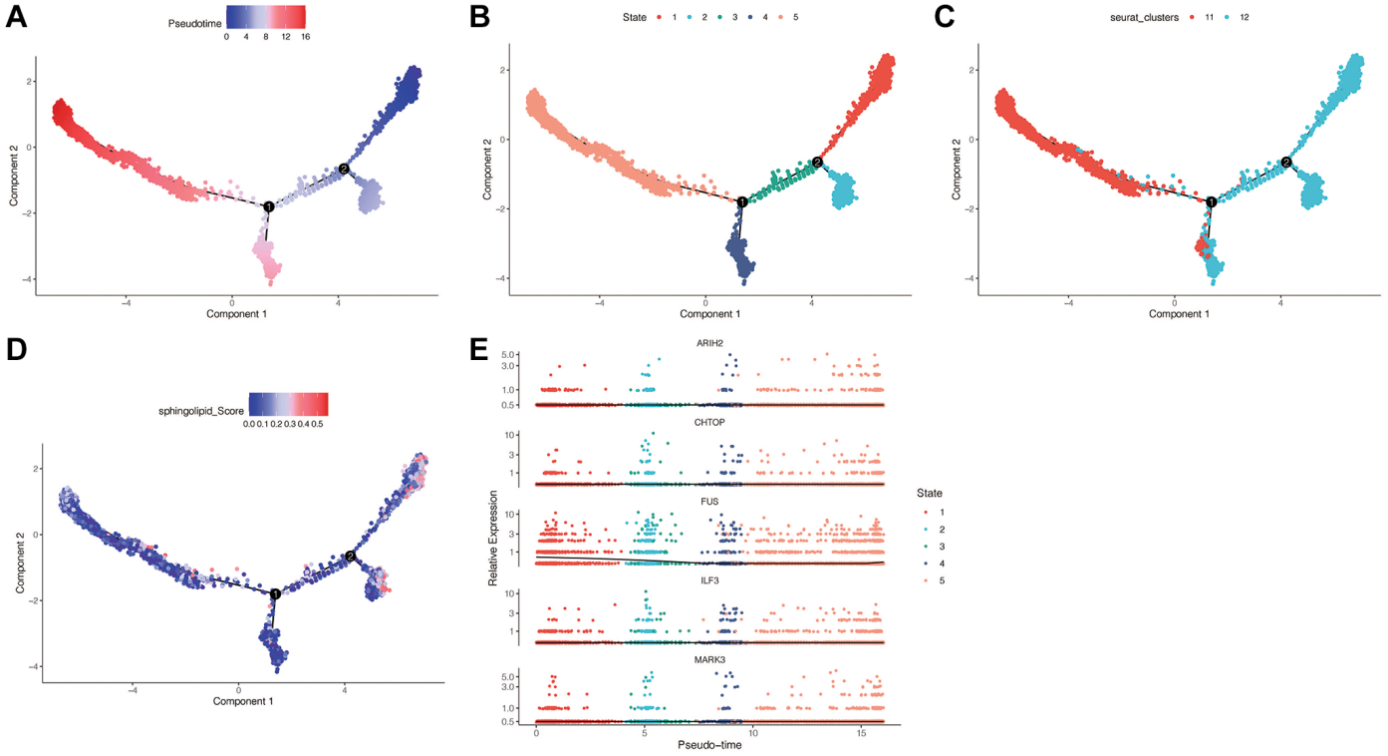
**Pseudo-time series analysis.** (**A**) Darker blue indicates early cell differentiation, while darker red indicates late differentiation. (**B**) Different cell differentiation states in total. (**C**) Different clusters. (**D**) The relation between sphingolipid enrichment score and cell differentiation. (**E**) The expression of FUS genes is gradually down-regulated as Fibroblast Cells differentiate.

In Figure 5A–5D, the color scale indicates the differentiation stages, with darker blue representing early cell differentiation and darker red indicating late differentiation. There are five cell differentiation states in total, with cluster 12 primarily in differentiation states 1, 2, and 3, while cluster 11 primarily occupies state 1, and state 4 contains both cluster 11 and cluster 12. Additionally, we observed that Fibroblast Cells with high sphingolipid enrichment scores exhibited more advanced differentiation.

As displayed in Figure 5E, the expression of the FUS gene gradually down-regulates as Fibroblast Cells undergo differentiation.

### Immune cell infiltration and drug sensitivity analysis

**Figure 6:**
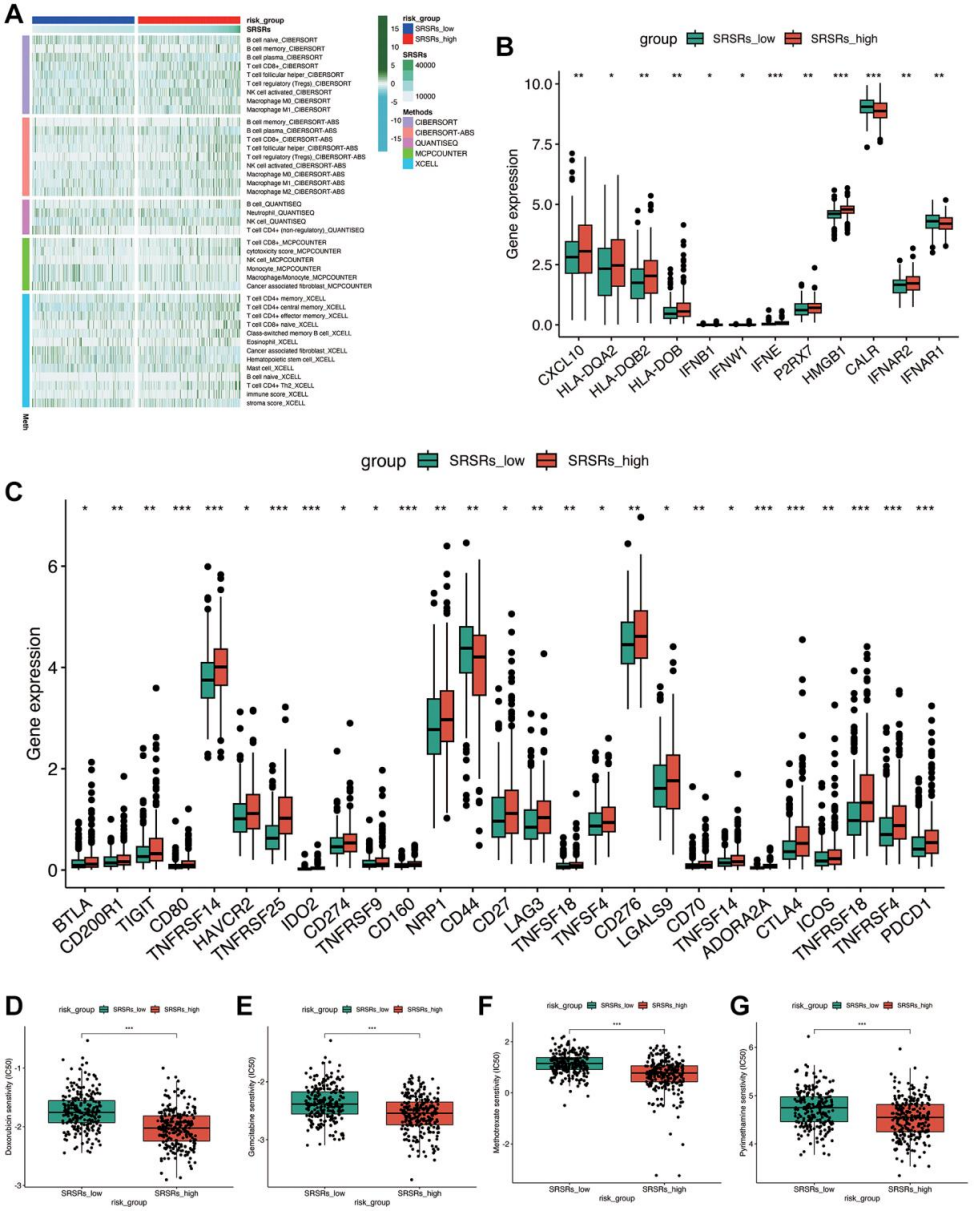
**Immune cell infiltration and drug sensitivity analysis.** (**A**) Immune cell infiltration landscape between SRSRs_high group and SRSRs_low group. (**B**, **C**) The expression level of leukocyte antigen genes, immune checkpoint genes and tumor necrosis genes between SRSRs_high group and SRSRs_low group. (**D**–**G**) Drug sensitivity analysis.

We proceeded to investigate the disparities in immune cell infiltration between the two groups defined by the model. As depicted in Figure 6A, the SRSRs_high group exhibited a higher level of immune infiltration by T cells and NK cells. In Figure 6B, 6C, it was observed that most leukocyte antigen genes, immune checkpoint genes, and tumor necrosis genes were highly expressed in the SRSRs_high group, including genes like BTLA, CD80, PDCD1, CXCL10, and HLA-DQA2. However, some genes such as CD44 and CALR were underexpressed in the SRSRs_high group.

To enhance the potential for more effective patient treatment, we predicted potentially beneficial drugs. Figure 6D–6G illustrates that Doxorubicin, Gemcitabine, Methotrexate, and Pyrimethamine had lower IC50 values in patients within the SRSRs_high group compared to those in the SRSRs_low group, suggesting that these drugs may be more suitable for treating patients in the SRSRs_high group.

### Construction of the nomogram

**Figure 7:**
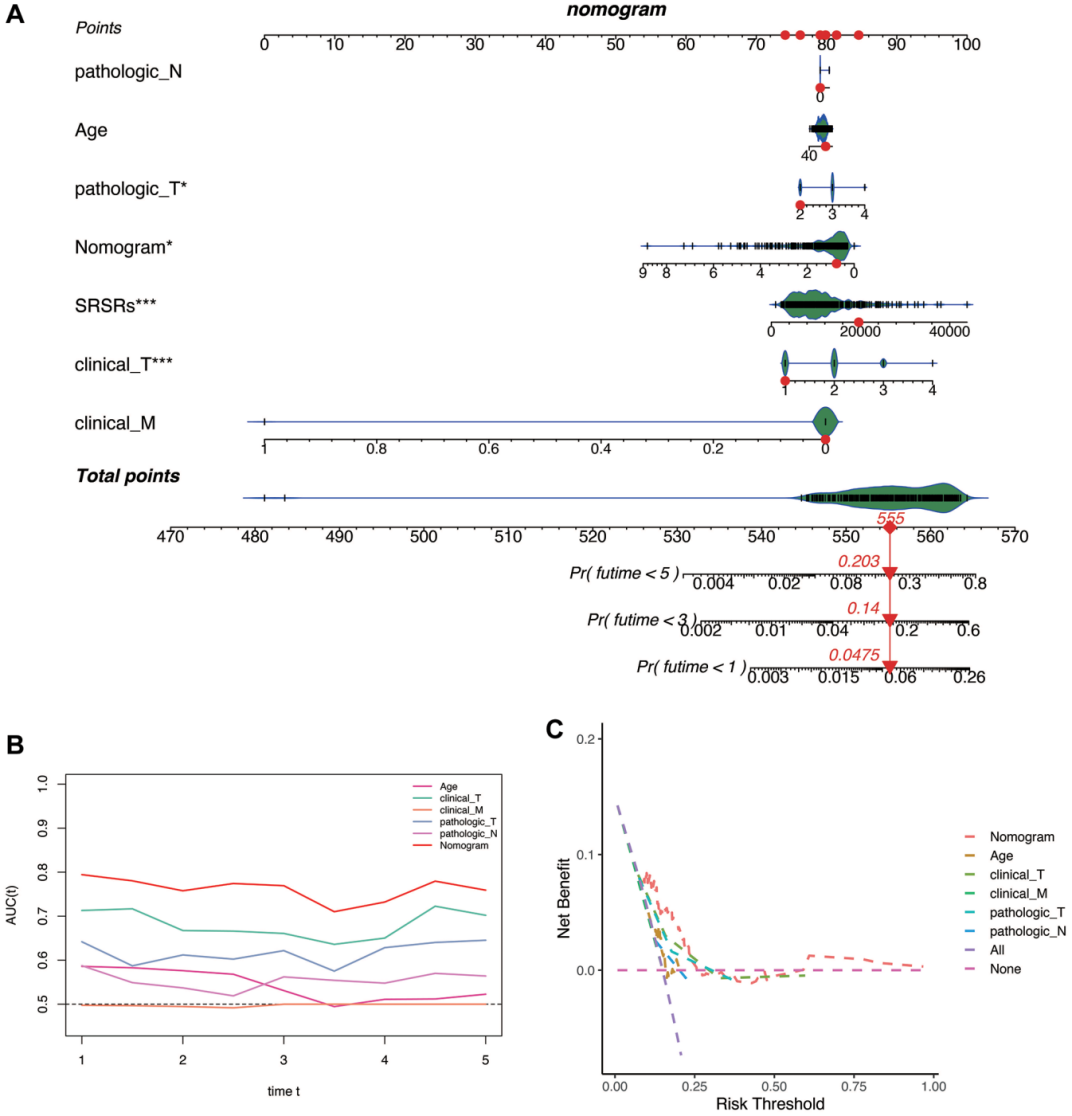
**Construction of the nomogram.** (**A**) The 1-, 3-, and 5-year recurrence or mortality rates for patients TCGA-HC-A8D0 in the TCGA database were 0.0475, 0.14, and 0.203. (**B**) Prognostic ROC curve analysis. (**C**) Clinical decision benefit curve.

To enhance the prediction of patient prognosis, we developed a nomogram. Figure 7A provides the 1, 3, and 5-year recurrence or mortality rates for patients in the TCGA database, which were 0.0475, 0.14, and 0.203, respectively. Figure 7B, 7C illustrate that the prognostic ROC curve analysis demonstrated the effectiveness of the nomogram in predicting patient prognosis, with an AUC consistently around 0.8. This performance surpassed that of other clinical indicators. Moreover, using the nomogram for clinical decision-making would be most advantageous for patients.

### PCR assay to verify the expression of ILF3 in prostate cancer tissues

**Figure 8:**
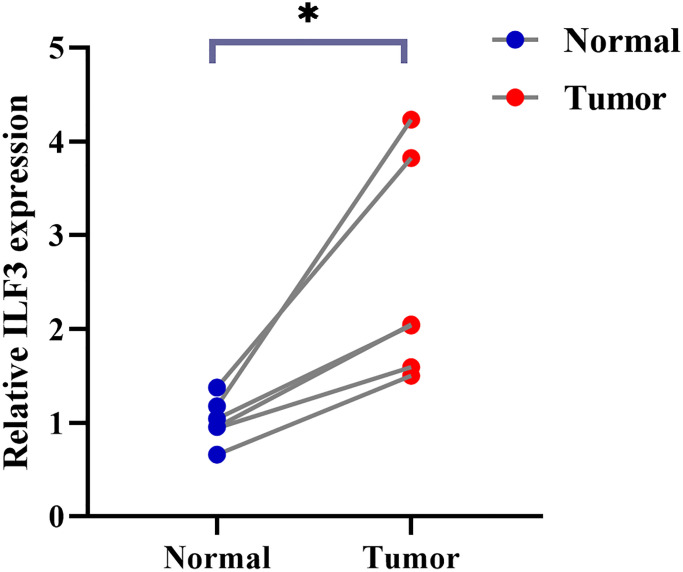
**PCR test.** ILF3 was highly expressed in prostate cancer tissues (^*^p < 0.05).

Figure 8 presents the results of PCR verification of ILF3 expression in prostate cancer tissues and normal controls. The results indicated a significant upregulation of ILF3 in prostate cancer tissues (^*^p < 0.05), suggesting its potential relevance in the context of prostate cancer.

## DISCUSSION

Our groundbreaking study integrates single-cell sequencing data with bulk transcriptome data to elucidate the significance of sphingoid metabolism in prostate cancer comprehensively. Through this innovative approach, we’ve unveiled the multifaceted roles of sphingoid metabolism, demonstrating its capacity for prognostic prediction, immune assessment, and drug sensitivity estimation in prostate cancer. Our investigation began with a deep dive into single-cell heterogeneity, followed by the identification of key sphingolipid metabolism-associated genes. This was complemented by bulk transcriptome analysis, leading to the development of a prognostic model comprising five crucial genes. Notably, our study explores the heterogeneity of gene expression in different cell types and states and investigates immune infiltration variations, highlighting the potential for immunotherapy. Furthermore, our findings suggest specific drugs, such as Doxorubicin, Gemcitabine, Methotrexate, and Pyrimethamine, as more effective for the high-risk group. Lastly, we constructed a predictive nomogram for prostate cancer patient prognosis. Overall, our research advances our understanding of prostate cancer biology and the role of sphingolipid metabolism, offering insights into diagnosis, treatment, and potential combined drug therapies.

Sphingolipid metabolism has emerged as a critical factor in cancer biology, offering valuable insights and therapeutic potential. Key sphingolipids like ceramides and sphingosine-1-phosphate (S1P) play contrasting roles as pro-apoptotic and pro-survival signaling molecules, respectively, influencing the fate of cancer cells. Dysregulation of sphingolipid metabolism is a common occurrence in various cancers and is linked to drug resistance. Researchers are actively exploring sphingolipid-related pathways as potential avenues for cancer therapy, including sphingosine kinase inhibitors and strategies to boost ceramide levels [22]. This evolving field holds promise for personalized cancer treatments and improved patient outcomes, underlining its current and future significance in cancer research.

Moreover, several bioinformatic analyses have shed light on the role of sphingolipid metabolism in cancer. For instance, Meshcheryakova et al. investigated the significance of sphingolipid metabolism in epithelial ovarian cancer, constructing survival models involving specific genes [23]. They also identified a novel network linking sphingolipids, lysophospholipids, and immune checkpoints, contributing to our understanding of tumor immune heterogeneity and disease outcomes. Similarly, Zhang et al. used various algorithms and RNA-seq data to pinpoint crucial genes in sphingolipid metabolism in lung adenocarcinoma, potentially opening new therapeutic avenues [24]. Our study, a pioneering effort, reveals the heterogeneity of sphingoid metabolism in prostate cancer and establishes a prognostic model, providing valuable tools for patient stratification and early intervention in prostate cancer.
